# An Add-On Treatment for Moderate COPD with Squill-Oxymel (a Traditional Formulation from *Drimia maritima (L.) Stearn*): A Pilot Randomized Triple-Blinded Placebo-Controlled Clinical Trial

**DOI:** 10.1155/2022/5024792

**Published:** 2022-05-30

**Authors:** Maryam Mohammadi–Araghi, Alireza Eslaminejad, Hossein Karegar-Borzi, Saeideh Mazloomzadeh, Fatemeh Nejatbakhsh

**Affiliations:** ^1^Department of Traditional Medicine, School of Persian Medicine, Tehran University of Medical Sciences, Tehran, Iran; ^2^Chronic Respiratory Diseases Research Center, National Research Institute of Tuberculosis and Lung Diseases (NRITLD), Shahid Beheshti University of Medical Sciences, Tehran, Iran; ^3^Neuroscience Research Center, Institute of Neuropharmacology, Kerman University of Medical Science, Kerman, Iran; ^4^Department of Traditional Medicine, Faculty of Persian Medicine, Kerman University of Medical Science, Kerman, Iran; ^5^Rajaie Cardiovascular Medical and Research Center, Iran University of Medical Sciences, Tehran, Iran; ^6^Food Microbiology Research Center, Tehran University of Medical Sciences, Tehran, Iran

## Abstract

**Background:**

In traditional Persian medicine, *Drimia maritima*, with the popular name Squill, has been used to alleviate phlegm dyspnea. Squill has also been shown to have anti-inflammatory and anticholinergic properties. The goal of this research was to see how effective and safe Squill-Oxymel was in treating COPD patients.

**Method:**

Forty-two COPD patients were examined for eight weeks in two groups. Patients underwent a 6-minute walk test to assess the treatment's effectiveness at the beginning and conclusion of the intervention. We utilized St. George's Respiratory Questionnaire (SGRQ) to evaluate the subjective symptoms of patients in order to measure their quality of life.

**Results:**

Patients who received Squill-Oxymel showed a statistically significant increase in 6MWT distance (*P*=0.011). The mean O_2_ saturation at the end of the 6MWT before the intervention was significantly greater in the placebo group. (*P*=0.008). In terms of questionnaire variables, there was a significant difference between placebo and Squill-Oxymel groups in the mean presymptom score (*P*=0.009) and the mean post-symptom score (*P*=0.004).

**Conclusions:**

The findings of this research provide preliminary evidence for the effectiveness and safety of Squill-Oxymel as an add-on therapy in individuals with mild COPD.

## 1. Introduction

Chronic obstructive pulmonary disease (COPD) is one of the major causes of death in the world [1].

The primary indication of this illness is chronic inflammation of the airway and lung parenchyma, which gradually and permanently reduces airflow [2]. The main symptoms of this disease are cough, sputum production, and dyspnea with exertion [3]. Based on the causes mentioned for dyspnea in Persian medicine, COPD symptoms are very similar to the type of dyspnea that is caused by the production of thick phlegm in the lungs [4–7]. One of the most important treatments for this type of dyspnea in Persian medicine is Squill-Oxymel [8, 9]. Squill, also known as *Drimia maritima (L.) Stearn*, was known to the Egyptians, Greeks, and Iranians for thousands of years, and they were well-versed in the medicinal applications of Squill and Squill-Oxymel [10], especially in the treatment of phlegm dyspnea [8, 9]. Nowadays, smoking cessation, oxygen delivery, and surgery are all effective COPD treatments. Glucocorticoids, bronchodilators, theophylline, and acetylcysteine are examples of common pharmacological treatments that merely alleviate symptoms and decrease the severity of the illness [1]. In general, treatment results are often lower than anticipated. On the other hand, several popular therapies are linked to side effects such as cardiovascular problems, oropharyngeal candidiasis, and pneumonia risk [1]. As a result, doctors are actively exploring safer alternatives to these medicines that are still effective [1]. Herbal medicines and their application in the treatment of different illnesses have gotten a lot of interest in recent years [11]. Because of the significance of COPD and the need for low-complication therapies, medicinal plants have gotten a lot of attention. For example, in a research by Boskabadi et al. in 2014, researchers looked at the effects of ginger extract (*Zataria multiflora*) on mice with COPD. The benefits of ginger hydroethanolic extract on all COPD parameters in these animals were shown to be equivalent to or even higher than dexamethasone in this research [12]. Various herbal formulations have been utilized in the treatment of respiratory illnesses accompanied by symptoms of sputum, cough, and dyspnea in traditional Persian medicine (TPM). In most cases, to achieve the desired clinical results, medicinal herbs are used in combination formulations [13]. Squill, particularly its component Sqill-Oxymel, is one of the most significant and extensively used medicines [4–7, 9, 14]. At present, Squill is applied in the manufacture of more than 70 drugs in reputable pharmaceutical companies worldwide, such as Pfizer and Sanofi Aventis [15].

Squill-Oxymel was able to control concentrated and sticky secretions [4–7, 9, 14] based on traditional Iranian medicine knowledge, and this pharmacological effect of Squill-Oxymel as well as absence of significant side effects has been confirmed in the study about the effectiveness of this medicine on asthmatic patients by Nejatbakhsh et al. On the other hand, it is much less expensive than existing therapies. Also, according to Zargaran et al., in a study published in 2012, Squill-Oxymel with the formulation of Squill, vinegar, and honey was useful and effective in respiratory diseases, asthma, and cough [17]. One of the ingredients used in Squill-Oxymel formulation is honey. Honey contains effective components such as flavonoids, phenolic acids, phenolic compounds, and terpenes and also has anti-inflammatory, antimicrobial, antioxidant, and immunomodulatory activities [18]. Saket et al., in a review article in 2020, showed that Squill-Oxymel has been used to treat many diseases, especially respiratory diseases [19]. Traditional Persian medicine as a branch of complementary and alternative medicine (CAM) plays a significant role in ensuring public health and controlling and treating diseases [20]. In order to establish the true position of this medicine alongside conventional medicine, there is a need for extensive evidence-based studies. Although research shows that Squill-Oxymel is an effective remedy for respiratory diseases, no research has been done on the effect of Squill-Oxymel on COPD patients. Therefore, the therapeutic effect of less than expected with routine drugs in COPD, the appropriate effect of Squill-Oxymel on asthma and absence of significant side effects with that according to clinical trial, lack of clinical study to determine the effect of Squill-Oxymel on COPD, and the emphasis of Persian medicine sources on the effectiveness of Squill-Oxymel on respiratory disease were all reasons for this study. This research was done to assess the impact of Squill-Oxymel on COPD.

## 2. Materials and Methods

### 2.1. Design

The efficacy of Squill-Oxymel in the treatment of patients with moderate to severe COPD was examined in this research, which was a placebo-controlled three-blind randomized clinical trial. To perform this study, the Research Ethical Committee of Tehran University of Medical Sciences has issued an ethics code with the following number: IR.TUMS.REC.1394.1092. The Declaration of Helsinki has also given its approval to the study [21]. The following is the IRCT registration code: IRCT2016040919912N2. This research was also sponsored financially by the Research Department of Tehran University of Medical Sciences, with the following number: 94-02-86-27538. Prior to enrolment, all patients gave their written permission, which was done ethically.

### 2.2. Sample Size Calculation

To identify the sample size, we applied the following formula:

(1) $$\begin{array}{c} n_{1}=\frac{{\left(Z_{1-\left(\alpha /2\right)}+Z_{1-\beta }\right)}^{2}\times \left(\sigma_{1}^{2}+\left(\sigma_{2}^{2}/k\right)\right)}{\Delta^{2}}. \end{array}$$

Applying the formula and following information on our primary outcome that was the 6-Minute Walking Test (6MWT), we calculated the number of samples required for every group. Our minimal detectable clinically important difference was 55 meters in the 6MWT.

(2) $$\begin{array}{c} \alpha =0.05⟶Z_{1-\left(\alpha /2\right)}=1.96,\\ \beta =0.20⟶Z_{1-\beta }=0.85,\\ \sigma_{1}=\sigma_{2}=60\text{ meters,}\\ k=1,\\ \Delta =\left|\mu_{1}-\mu_{2}\right|=\left|105-50\right|=55\text{ meters,} \end{array}$$

*n* = 19 and with 10 percent loss, *n* = 21.

### 2.3. Patients

COPD patients with moderate (Stage IIA) disease who were referred to the outpatient pulmonary clinic of Dr. Masih Daneshvari hospital, affiliated with Shahid Beheshti University of Medical Sciences, Tehran, Iran, between May 2019 and May 2020 and met the inclusion criteria and signed an informed consent form were included in this study. One of the important indicators for the diagnosis and staging of COPD patients is spirometry. Forced expiratory volume in 1 s (FEV1)/forced vital capacity (FVC) ratio of <0.7 is one of the important points in the diagnosis of COPD. The Global Initiative for Lung Disease (GOLD) recommendations were used to determine the diagnostic criteria for moderate (Stage IIA) COPD, which included forced expiratory volume in 1 s (FEV1)/forced vital capacity (FVC) ratio of < 0.7, 50% ≤ FEV1 < 80% of predicted [3]. Patients aged 40–80 years [22], diagnosed with COPD by a pulmonologist, being treated with conventional drugs, mild COPD patients (Stage IIA), no pregnancy, no nursing, nonconcurrent use of other herbal medicines, and no history of allergy to herbal medicines were all included in the study. Patients with no active peptic ulcer, no gastrointestinal symptoms such as pain, heartburn, or reflux, no known cardiovascular illness (particularly heart block) or renal disease, and not using anticoagulants concurrently were excluded from the study. The use of medications that interfere with COPD therapy, such as NSAIDs and corticosteroids, recent infections resulting in hospitalization, or the patient's reluctance to continue treatment were also exclusion factors.

### 2.4. Plant Analysis

*Drimia maritima* was collected in Kazerun city which is situated at an altitude of about 1050 meters in the Fars region, Iran. The Faculty of Pharmacy's herbarium has a voucher specimen (No. 6622-TEH). Bufadienolides are one of the plant's major photochemical substances. Stoll et al. identified proscillaridin A, the first bufadienolide chemical, in 1933 [23]. The bufadienolides in Squill were quantified using high-performance liquid chromatography (HPLC). Methanol was the best solvent for extracting proscillaridin A because it had a greater recovery rate and caused less interference than other solvents [24].

Researchers used 10 *µ*l of the test solution in triplicate on a TLC plate. Then, they developed the plate in the solvent system to a distance of 8 cm and recorded the chromatogram and area under the curve for proscillaridin A as explained under the calibration curve. At last, they identified the amount of proscillaridin A in the sample by applying the calibration curve of proscillaridin A. The amount of proscillaridin A per each gram of the plant powder was 1 mg. [16].

### 2.5. Preparation of Syrup and Placebo

Professor Nazem E. of the Department of Traditional Pharmacy at the Faculty of Traditional Medicine, Tehran University of Medical Sciences, Tehran, Iran, oversaw the manufacturing of this medicine (Squill-Oxymel). The medication was made according to the patterns described in the traditional medical literature. When cleaning and washing the Squill rhizomes, we break them into pieces and boil them in vinegar and then add honey after the bulbs have softened. The quantity of acetic acid in this medication was 2.24–2.4 percent w/v, according to British Pharmacopoeia's standards [25]. The placebo was made from honey that had been cooked in water. The containers for the Squill-Oxymel syrup and the placebo were identical.

### 2.6. Protocol

The current study was a randomized, double-blind, placebo-controlled experiment. Eligible individuals were randomly assigned to the control group (arm B) and the therapeutic group (Squill-Oxymel = arm A). Random permuted blocks of size 4 were used for randomization and a random allocation sequence was created by a random-numbers table. The allocated intervention was kept a secret from all patients, researchers, and assessors. Following enrolment in the trial, patients were given either Squill-Oxymel or a placebo as an adjuvant at a dosage of 10 ml twice daily for four weeks, depending on the group they were assigned to. The medicines were administered to the patients by a single nurse who was likewise unaware of the study's existence. Researchers at the University of Tehran's Department of Traditional Pharmacy produced Squill-Oxymel and placebo in the form of syrup. The instructions to take the medicines are as follows: take it twice a day, once in the morning while fasting and again before bedtime, at a dosage of 10 ml each time. It should be emphasized that the aforementioned medicines are being added to the above patients' regular therapies, and patients should not alter the quantity or manner of taking their prior medications. Patients must visit the clinic at weeks 4 and 8, and the amount of medication is divided based on these times and given to patients each time.

Hence, the questionnaire completion and the 6-minute walking test were only done at the beginning and end of the intervention.

### 2.7. 6-Minute Walking Test

The 6-minutes walking test (6MWT) is a simple and safe test that, in comparison to previous walking tests, is more tolerable and more accurately represents everyday activity. The 6-minute walk test (6MWT) has been defined by the American Thoracic Society [26]. This test was performed twice: first at the start of the trial and again after 8 weeks of therapy. The patient's blood oxygen saturation and feeling of dyspnea may also be evaluated throughout the test. The distance walked in 6 minutes is the first factor examined in this test. No one should help her (him) walking, and the patient's gaiting should be alone. It is also against the rules to walk on a treadmill and alter the pace or inclination. Walking on an oval or circular route is not recommended. In the 6MWT, reassurance and enthusiasm may make a 30 percent impact [26].

### 2.8. St. George's Respiratory Questionnaire (SGRQ)

Nowadays, physical, psychological, and social function of individuals plays an important role in choosing the appropriate treatment method and controlling chronic diseases [27]. St. George's Questionnaire examines the effect of the treatment on improving patients' lifestyles.

The SGRQ is a standardized, self-administered questionnaire for assessing the quality of life (QOL) in individuals with chronic obstructive pulmonary disease (COPD). The SGRQ's total score included the frequency and severity of respiratory symptoms, activity limitations caused by dyspnea, and social and psychological factors. As a result, the total score sums up the disease's impact on overall health. Patients can understand it easily. Higher scores are associated with a worse quality of life in terms of health [28]. The overall score of St. George's Respiratory Questionnaire is assessed at the beginning and 8 weeks after the commencement of the research to measure the patient's quality of life. This questionnaire was translated into Persian several years ago. However, the validity and reliability of its Persian version were evaluated by Mr. Tafti et al. in 2014 and were confirmed with good results [29].

### 2.9. Statistical Analysis

The Kolmogorov–Smirnov test was used to evaluate the distribution of quantitative variables. Values were expressed as numbers (percentage) and mean ± standard deviation, as appropriate. Comparisons were performed by the Fisher exact test for categorical variables, independent or the paired *t*-test for normally distributed variables, and Mann–Whitney or Wilcoxon signed-rank test for nonnormally distributed variables. All statistical analyses were performed using Statistical Package IBM SPSS Statistics for Windows, version 22 (IBM Corp., Armonk, NY, USA).

## 3. Results

**Figure 1:**
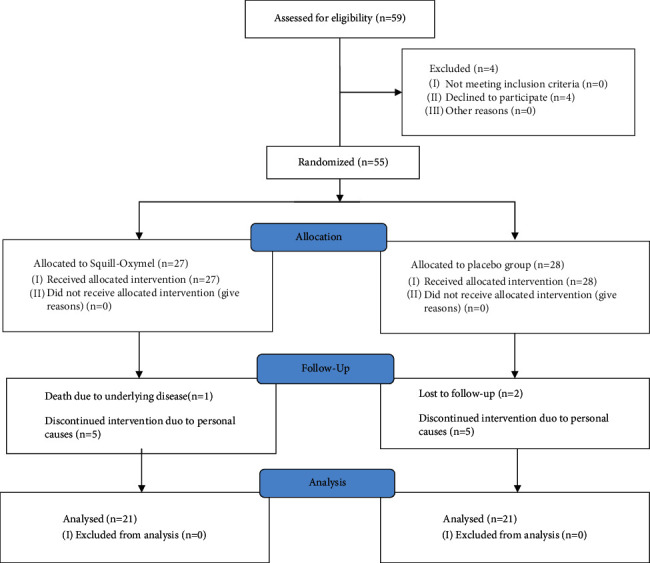
Registration, randomization, and patient outcome.

There were 59 patients in total that were referred to the research. They were randomized to the therapeutic and control groups. During the follow-up, nine patients from each group were dropped from the trial.  Figure 1 shows the registration, randomization, and patient outcome explanations.

Patients in the Squill-Oxymel group were 60.00 ± 8.99 years old, whereas those in the placebo group were 61.10 ± 7.78 years old (*P* = 0.68, Table 1). The proportion of men in the Squill-Oxymel group was 47.4% and in placebo group was 52.6% (*P* = 0.61, Table 1).

The mean O_2_ saturation at the end of the 6MWT before the intervention was 85.57 in the placebo group and 77.00 in the Squill-Oxymel group, and the difference was statistically significant (*P*=0.008, Table 2).

The mean distance traveling in the 6MWT in the Squill-Oxymel group was 369.76 before the intervention and 387.38 after the intervention and the difference was statistically significant (*P*=0.011, Table 3). The mean O_2_ saturation at the end of the 6MWT in the placebo group was 85.57 before the intervention and 81.29 after the intervention (*P*=0.003, Table 3). However, other dimensions of the 6MWT were not statistically different between Squill-Oxymel and placebo groups.

The mean St. George's Questionnaire symptom score before the intervention was 42.64 in the placebo group and 61.19 in the Squill-Oxymel group, and the difference was statistically significant (*P*=0.009, Table 4). The mean St. George's Questionnaire symptom score after the intervention was 36.69 in the placebo group and 56.53 in the Squill-Oxymel group (*P*=0.004). However, the total score and other dimensions of St. George's Questionnaire were not statistically different between Squill-Oxymel and placebo groups.

Before and after the intervention, the mean scores of St. George's symptom were 42.64 and 36.69 in the placebo group and 61.19 and 56.53 in the Squill-Oxymel group, respectively (Table 5). The reduction was statistically significant in the placebo group (*P*=0.01). The total score and other dimensions of St. George's Questionnaire were not statistically different before and after the intervention in none of the two groups.

## 4. Discussion

The selection of the right treatment depends on two factors: severity of symptoms and frequency of exacerbations [30].

Inhaling bronchodilators is the most often prescribed treatment for stable COPD symptoms. Triple treatment (inhaled glucocorticoids + long-acting b2-agonist + long-acting muscarinic receptor antagonist) is being considered in certain instances to prevent the illness from worsening and to improve work capacity. However, in many instances, these medicines are unable to halt the illness from progressing [1]. Despite the numerous difficulties surrounding the use of 2-adrenoceptor agonists, long-acting-agonists (LABAs) and long-acting muscarinic antagonists (LAMAs) are the primary treatments in patients with stable COPD [31–33]. However, these medicines may induce cardiac issues in COPD patients, particularly in those who already have a heart condition [31]. Furthermore, a review and meta-analysis of nine clinical studies found that LABAs alone had little effect on COPD patients' death rates [31]. The most significant pathological alteration in COPD is inflammation of the airways, which is why glucocorticoids are used to treat it [2]. Applying inhaled corticosteroids (ICSs) in long-term treatment of COPD is controversial [34].

Long-term use of this medication has been linked to a number of negative side effects. These adverse effects are less frequent than those observed in individuals using systemic corticosteroids, but they include diabetes, cataracts, osteoporosis, adrenal insufficiency, active TB, skin bruising, and electrolyte imbalance [34]. Oral phosphodiesterase 4 (PDE4) inhibitors such as roflumilast and cilomilast are used to decrease airway inflammation and bronchoconstriction [35]. PDE4 inhibitors were shown to have a greater impact on improving lung function and decreasing exacerbation than placebo in one trial, but they had no effect on enhancing quality of life or lowering symptoms. Weight loss and gastrointestinal adverse effects have also been observed. According to the US Food and Drug Administration (FDA), concerns regarding roflumilast's mental adverse effects are increasing [35].

Traditional Persian medicine (TPM) has utilized Squill-Oxymel, a Squill-based medicinal substance, to treat a variety of illnesses including melancholia, paralysis, dyspepsia, flatulence, jaundice, ascites, sciatica, and arthritis [16]. Respiratory issues such as hoarseness, pneumonia, chronic bronchitis, chronic cough, and asthma have all been treated with this medication [6, 36, 37]. Squill contains anti-inflammatory, antioxidant, antibacterial, anticholinergic, and mucus production regulating properties, according to the information obtained [38–41]. The anticholinergic and Ca2+ antagonistic properties of crude extract of Indian Squill *(Drimia indica*) are likely responsible for its bronchodilator (broncho-relaxant) properties. Indian Squill's muscarinic (m3 receptors) antagonist action also reduces mucus production in the airway [42]. This medication may be utilized in patients with concentrated respiratory secretions, according to Iranian medical principles and recent discoveries. It has been utilized to treat phlegm dyspnea in particular [9, 14]. Squill-Oxymel formulation is also registered in British Pharmacopoeia [25].

According to the findings, patients who received Squill-Oxymel showed a statistically significant increase in the 6MWT distance (*P*=0.011, Table 3). Furthermore, in the Squill-Oxymel group, there was no significant change in the mean score of any of the criteria or the total score before and after the intervention. The placebo group had a significantly greater blood oxygen saturation at the conclusion of the 6MWT than the Squill-Oxymel group before starting the intervention (*P*=0.008, Table 2). Accordingly, illness severity in the placebo group at the beginning of the study was lower than that of the Squill-Oxymel group. Also, the symptom score in the Squill-Oxymel group before starting the intervention was significantly greater than that of the placebo group (*P*=0.009, Table 4). This also indicates that the disease is more severe in the Squill-Oxymel group. Furthermore, in the placebo group, the symptom score was significantly less than the Squill-Oxymel group both before and after the intervention, and it indicates that generally, the severity of symptoms was lower in the placebo group (*P*=0.009, *P*=0.004, Table 4). Despite the intensity of symptoms in the Squill-Oxymel group, the average distance traveled in the 6MWT in this group rose after the intervention compared to before the intervention. The symptom mean ratings in the placebo group were 42.64 and 36.69 before and after the intervention, respectively, indicating a significant reduction (*P*=0.01, Table 5). Perhaps this reduction in the symptoms score in the placebo group was due to the anti-inflammatory effect of honey which was used in the placebo. In other words, considering that the severity of symptoms in the placebo group was lower than that in the Squill-Oxymel group before the intervention, the effect of honey on improving the symptoms of patients in the placebo group was clear and significant. In fact, the intensity of symptoms decreased in both Squill-Oxymel and placebo groups, although it decreased significantly in the placebo group. On the other hand, blood oxygen saturation at the end of the 6MWT after the intervention significantly decreased in the placebo group (*P*=0.003, Table 3) and it shows that despite the less severity of symptoms in this group, their respiratory condition has worsened at the end of the study than the beginning of the study. As a result of this research, Squill-Oxymel may help these individuals' respiratory health.

There is evidence for the anti-inflammatory and anticholinergic impacts of Squill [16].

The primary components of Squill that have immunomodulatory effects are bufadienolides. They demonstrate this activity by inhibiting the activation of peripheral T cells. They have a far greater ability to regulate the immune system than steroids and other immunosuppressive drugs [16]. Persistent inflammation of the airway and lung parenchyma is the primary sign of this illness, which causes symptoms such as chronic cough, sputum, and dyspnea [2]. To decrease inflammation, inhaled corticosteroids (ICS) are presently utilized. However, their usage is restricted owing to adverse effects such as a higher risk of pneumonia in patients [16], diabetes, cataracts, osteoporosis, adrenal insufficiency, active tuberculosis, skin bruising, and electrolyte imbalance [34]. Furthermore, unlike asthmatics, COPD patients do not react well to steroid treatment [43]. Increased mucus production, on the other hand, is one of the most common reasons for airway blockage in COPD patients [43]. Inflammation of the respiratory system in these individuals, according to traditional Iranian medicine (TIM), implies thick phlegm, which indicates mucus hypersecretion in the lungs and airways. Due to the softening of existing phlegm, Squill-Oxymel may help patients with their symptoms [4]. Furthermore, no ICS-related problems were observed in individuals treated with Squill-Oxymel during regular follow-up.

Indian Squill possesses a smooth muscle relaxant action in the respiratory tract, according to Bashir et al. [42]; with calcium influx blocking and anticholinergic effects. COPD causes a little contraction and an increase in the mass of the airway smooth muscle [43]. As a result, it seems that raising blood oxygen saturation and distance walked in the 6MWT has a limited relationship with Squill's anticholinergic impact and is more closely linked to its anti-inflammatory effect. An increase in oxidative stress, which is worsened endogenously or by a reduction in antioxidants in the diet, is one of the mechanisms in the pathophysiology of COPD [43]. Squill has antioxidant action, according to Mammadov et al. [44]. During the research, no severe adverse effects were identified. No patient experienced symptoms suggestive of adrenal insufficiency or corticosteroid side effects during regular outpatient follow-up after the trial ended.

This study has two limitations. The first is the small sample size. Our study was the first clinical trial on Squill-Oxymel in COPD patients. Subsequent clinical trials with a larger sample size are necessary for the assessment of its efficacy and safety. The second is that the honey in placebo has an anti-inflammatory effect and this can distort the result of the study.

## 5. Conclusion

The findings of this research provide preliminary evidence for the effectiveness and safety of Squill-Oxymel as an add-on therapy in individuals with mild COPD. For the findings to be confirmed, further research with bigger sample numbers and longer follow-up periods is required.

## Figures and Tables

**Table 1:** Characteristics of the study population by the groups.

Variables	Squill-Oxymel group (*n* = 21)	Placebo group (*n* = 21)	*P*
Age (mean ± SD)	60.00 ± 8.99	61.10 ± 7.78	0.68
Sex, male, *n* (%)	18 (47.4%)	20 (52.6%)	0.61

**Table 2:** Comparison of the mean distance in the 6MWT and O_2_ saturation between Squill-Oxymel and placebo groups.

Variables	Squill-Oxymel group M ± SD	Placebo group M ± SD	*P* Mann–Whitney *U* test
Predistance in the 6MWT Postdistance in the 6MWT	369.76 ± 117.26 387.38 ± 123.53	410.00 ± 133.33 399.81 ± 106.40	0.199 0.940

Pre-O_2_ saturation at the start of the 6MWT Post-O_2_ saturation at the start of the 6MWT	86.90 ± 8.64 85.19 ± 11.25	89.95 ± 5.40 88.90 ± 6.56	0.30 0.37

Pre-O_2_ saturation at the end of the 6MWT Post-O_2_ saturation at the end of the 6MWT	77.00 ± 13.28 77.86 ± 12.52	85.57 ± 9.10 81.29 ± 10.32	0.008 0.48

M ± SD: mean ± standard deviation.

**Table 3:** Comparison of the mean distance in the 6MWT and O_2_ saturation between Squill-Oxymel and placebo groups.

Variables	Group	Before M ± SD	After M ± SD	*P* Wilcoxon signed-rank test
Distance in the 6MWT	Squill-Oxymel Placebo	369.76 ± 117.26 410.00 ± 133.33	387.38 ± 123.53 399.81 ± 106.40	0.011 0.198

O_2_ saturation at start of the 6MWT	Squill-Oxymel Placebo	86.90 ± 8.64 89.95 ± 5.40	85.19 ± 11.25 88.90 ± 6.56	0.75 0.59

O_2_ saturation at end of the 6MWT	Squill-Oxymel Placebo	77.00 ± 13.28 85.57 ± 9.10	77.86 ± 12.52 81.29 ± 10.32	0.37 0.003

M ± SD: mean ± standard deviation.

**Table 4:** Comparison of the mean score of total and dimensions of St. George's Questionnaire between Squill-Oxymel and placebo groups.

Variables	Squill-Oxymel group M ± SD	Placebo group M ± SD	*P* independent *t*-test
Pre-St. George's symptom score Post-St. George's symptom score	61.19 ± 22.98 56.53 ± 25.04	42.64 ± 20.55 36.69 ± 16.84	0.009 0.004

Pre-St. George's activity score Post-St. George's activity score	61.77 ± 22.32 59.52 ± 22.18	53.97 ± 24.42 55.59 ± 26.01	0.29 0.60

Pre-St. George's impact score Post-St. George's impact score	39.06 ± 24.45 38.89 ± 24.31	33.05 ± 22.88 31.96 ± 24.35	0.42 0.36

Pre-St. George's total score Post-St. George's total score	49.65 ± 21.44 48.21 ± 21.92	41.01 ± 21.21 40.00 ± 22.03	0.20 0.23

M ± SD: mean ± standard deviation.

**Table 5:** Comparison of the mean score of total and dimensions of St. George's questionnaire between Squill-Oxymel and placebo groups.

Variables	Group	Before M ± S—	After M ± S—	*P*paired *t*-test
St. George symptom score	Squill-Oxymel Placebo	61.19 ± 22.97691 42.64 ± 20.54949	56.53 ± 25.04019 36.69 ± 16.84810	0.24 0.01

St. George's activity score	Squill-Oxymel Placebo	61.77 ± 22.31835 53.97 ± 24.42456	59.52 ± 22.17675 55.59 ± 26.01322	0.22 0.41

St. George's impact score	Squill-Oxymel Placebo	39.06 ± 24.44876 33.05 ± 22.87777	38.89 ± 24.31328 31.96 ± 24.34633	0.95 0.66

St. George's total score	Squill-Oxymel Placebo	49.65 ± 21.43897 41.01 ± 21.20870	48.21 ± 21.92016 40.00 ± 22.03344	0.55 0.50

M ± SD: mean ± standard deviation.

## Data Availability

The data used to support the findings of this study are available from the corresponding author upon request.
